# Immune reconstitution inflammatory syndrome‐associated lymphoma: A retrospective Brazilian cohort

**DOI:** 10.1002/jha2.835

**Published:** 2023-12-20

**Authors:** Juliano Cordova Vargas, Karin Zattar Cecyn, Mariana de Oliveira Marques, Juliana Pereira, Walter M. Tobias Braga, Nelson Hamerschlak, Jacques Tabacof, Caina D. de Liz, André Abdo, Paulo Roberto Abrão Ferreira, Gisele W. Braga Colleoni, Otavio C. G. Baiocchi

**Affiliations:** ^1^ Department of Clinical and Experimental Oncology Federal University of São Paulo São Paulo SP Brazil; ^2^ Department of Hematology Américas Oncologia e Hematologia São Paulo SP Brazil; ^3^ School of Medicine Centro Universitário São Camilo São Paulo SP Brazil; ^4^ Department of Hematology Hospital Alemão Oswaldo Cruz São Paulo SP Brazil; ^5^ Department of Clinical and Hematology São Paulo State Cancer Institute University of São Paulo São Paulo SP Brazil; ^6^ Department of Hematology Emílio Ribas Institute of Infectious Diseases São Paulo SP Brazil; ^7^ Department of Hematology Hospital Israelita Albert Einstein São Paulo SP Brazil; ^8^ Centro Paulista de Oncologia São Paulo SP Brazil; ^9^ Department of Hematology Hospital Sírio Libanês São Paulo SP Brazil; ^10^ Division of Infectious Disease Escola Paulista de Medicina Federal University of São Paulo São Paulo SP Brazil

**Keywords:** antiretroviral therapy, immune reconstitution inflammatory syndrome, lymphoma

## Abstract

After initiating combined antiretroviral therapy (cART), individuals with human immunodeficiency virus (HIV) may develop Hodgkin/non‐Hodgkin lymphoma due to immune reconstitution inflammatory syndrome (IRIS). This retrospective cohort study evaluated the incidence, clinical features and prognosis of IRIS‐associated lymphomas in Brazilian patients. Incidence in 2000–2019 was 9.8% (27/276 patients with HIV and lymphoma; viral load drop >1 log). Time between HIV diagnosis and cART initiation was <1 year in 70.3% of cases. Time between cART initiation and lymphoma diagnosis was <3 months in 11 cases and 3–6 months in 16 cases. Overall and progression‐free survival rates were similar between cases of non‐IRIS‐associated lymphoma and IRIS‐associated lymphoma.

**Abbreviations**
cART: combined antiretroviral therapy
EBV: Epstein–Barr virus
HIV: human immunodeficiency virus
HL: Hodgkin lymphoma
IRIS: immune reconstitution inflammatory syndrome
NHL: non‐Hodgkin lymphoma

## 1 BACKGROUND

Cancer, particularly lymphoma, is the leading cause of death in people living with human immunodeficiency virus (HIV) in the combined antiretroviral therapy (cART) era [1, 2, 3]. During the first 6 months of cART use, individuals with HIV are at an increased risk of developing Hodgkin/non‐Hodgkin lymphoma (HL/NHL), probably due to immune reconstitution inflammatory syndrome (IRIS), previously described in cases of opportunistic infections and Kaposi sarcoma [1, 2, 4]. The hallmark of IRIS is paradoxical worsening of an existing infection/disease or the appearance of a new condition shortly after initiating cART, together with a viral load drop ≥1 log and a concomitant increase in CD4 count [5]. The causes behind IRIS‐associated lymphomas remain unclear [3], and data on the clinical characteristics and outcomes are sparse.

This study evaluated the incidence, clinical features and prognosis of IRIS‐associated lymphoma in the largest Brazilian cohort of HIV‐infected patients receiving cART.

## 2 METHODS

This retrospective, multicentre, observational study was conducted between 2000 and 2019 in the city of São Paulo, Brazil. Inclusion criteria comprised age >18 years, living with HIV and a biopsy‐confirmed lymphoma diagnosis based on the 2016 World Health Organization criteria [6]. Data on sex, age at diagnosis, Ann Arbor clinical stage, laboratory results, chemotherapy protocol and treatment response were retrieved from the medical charts. The response criteria were assessed in accordance with the 2007 Cheson criteria [7]. IRIS‐associated lymphoma was defined as HL/NHL diagnosed within 6 months of initiating cART. Viral suppression was defined as a reduction in the HIV RNA viral load ≥1 log_10_ copies/mL [5].

Statistical analysis was performed using SPSS for Windows (version 20.0). Frequencies and percentages (categorical variables) were compared using the chi‐square test or Fisher's exact test. Medians and interquartile ranges (non‐normal distribution) were compared using the Wilcoxon test or the Mann–Whitney test. Overall survival was calculated from the date of lymphoma diagnosis until administrative censoring, death or loss to follow‐up, while progression‐free survival was calculated from lymphoma diagnosis until imaging and biopsy‐documented relapse. Survival probabilities were estimated using the Kaplan–Meier method.

## 3 RESULTS

This cohort consisted of 276 patients with HIV and lymphoma, with 27 patients (9.8%) meeting the criteria for IRIS‐associated lymphoma. Most patients were male (*n* = 23, 85%). Median age was 42 years (range 25–62 years). In 21 patients (78%), Eastern Cooperative Oncology Group performance status score was 0 or 1. The time between HIV diagnosis and cART initiation was <1 year in 70.3% cases, 1–5 years in 14.8% cases and 6–15 years in 14.8% cases. At the time of lymphoma diagnosis, all the 27 patients were on cART, mostly nucleoside analogues and protease inhibitors, such as combination therapy with lamivudine and/or with tenofovir. The time between cART initiation and lymphoma diagnosis was <3 months in 11 cases (40.7%) and 3–6 months in 16 cases (59.3%).

At diagnosis of HIV infection (before starting cART), 21 patients (77.7%) had CD4 count <200 cells/mm^3^, two (7.4%) had 200–350 cells/mm^3^ and four (18.4%) had >350 cells/mm^3^. At lymphoma diagnosis, CD4 count was <200 cells/mm^3^ in 16 patients (59.2%), 200–350 cells/mm^3^ in six patients (22.2%) and >350 cells/mm^3^ in five patients (18.5%). Viral load at lymphoma diagnosis was <500 copies/mL in 16 cases (59.3%), 500–4999 copies/mL in three cases (11.1%), 5000–49,000 copies/mL in five cases (18.5%) and ≥50,000 copies/mL in three cases (11.1%). No cases of hepatitis B or C virus co‐infection were identified. One patient (3.7%) had an HL and the remaining 26 (96.2%) had NHL, with 16 of these (61.5%) having diffuse large B‐cell lymphoma, eight (33.4%) Burkitt lymphoma, one (2.5%) plasmablastic lymphoma and one (2.5%) anaplastic T‐cell lymphoma. In 20 cases (74.0%), lymphomas were at an advanced stage. Extranodal involvement was present in 15 cases (55.5%), which is as follows: bone marrow (*n* = 4, 26.0%), liver (*n* = 3, 20.0%), central nervous system meninges or parenchyma (*n* = 3, 20.0%), gastrointestinal tract (*n* = 3, 20.0%) and lung (*n* = 2, 14.0%). Chemotherapy regimens varied (Table 1).

**TABLE 1:** Characteristics of a cohort of human immunodeficiency virus (HIV)‐infected adults with immune reconstitution inflammatory syndrome‐associated lymphoma (2000–2019).

Characteristics	Number of patients, total *N* = 27
ECOG 0 or 1, *n* (%)	21 (78)
Median age (years)	42 (range 25–62 years)
Males, *n* (%)	23 (85)
Time between diagnosis of HIV and initiation of cART, *n* (%)	
<1 year	19 (70.3)
1–5 years	4 (14.8)
6–15 years	4 (14.8)
Time between initiation of cART and diagnosis of lymphoma, *n* (%)	
< 3 months	11 (47)
3–6 months	16 (59.3)
Histological type of lymphoma (%)	
Non‐Hodgkin lymphoma	96.2
Diffuse large B‐cell lymphoma	61.5
Burkitt lymphoma	33.4
Plasmablastic lymphoma	2.55
T‐cell lymphoma	2.55
Hodgkin lymphoma	3.7
Ki67 ≥90%, *n* (%)	21 (77)
Advanced stage, *n* (%)	20 (74)
Extranodal involvement, *n* (%)	15 (55)
Liver	3 (20)
Bone marrow	4 (26)
Central nervous system	3 (20)
Gastrointestinal tract	3 (20)
Lung	2 (14)
Chemotherapy protocol, *n* (%)	
CHOP	11 (40.7)
R‐CHOP	2 (7.4)
ABVD	1 (3.7)
CHOEP	4 (14.8)
DA‐EPOCH	3 (11.1)
Hyper‐CVAD	5 (18.5)
Codox‐M‐IVAC	1 (3.7)
Nadir CD4^+^ T lymphocyte count prior to initiation of cART, *n* (%)	
<200 cells/mm^3^	21 (77.7)
200–350 cells/mm^3^	2 (7.4)
>350 cells/mm^3^	4 (14.8)
Nadir CD4^+^ T lymphocyte count at diagnosis of lymphoma, *n* (%)	
<200 cells/mm^3^	16 (59.2)
200–350 cells/mm^3^	6 (22.2)
>350 cells/mm^3^	5 (18.5)
Detectable viral load at diagnosis of lymphoma, *n* (%)	
<500 copies/mL	16 (59.3)
500–4999 copies/mL	3 (11.1)
5000–49,000 copies/mL	5 (18.5)
>50,000 copies/mL	3 (11.1)
cART at the moment of the diagnosis of lymphoma, *n* (%)	
Protease inhibitor	1 (3.7)
NNRTI	6 (22.2)
NRTI	19 (70.3)
Other	1 (3.7)
Co‐infection with hepatitis B/C	None
Primary refractory, *n* (%)	4 (14.8)
Mean progression‐free survival (months)	67.8
Mean overall survival (months)	77.6
Mortality, *n* (%)	6 (22.4)

Abbreviations: ABVD, doxorubicin, bleomycin, vinblastine and dacarbazine; cART, combined antiretroviral therapy; CHOEP, cyclophosphamide, doxorubicin, etoposide, vincristine and prednisone; CHOP, cyclophosphamide, doxorubicin, vincristine and prednisone; CODOX‐M‐IVAC, cyclophosphamide, vincristine, doxorubicin, high‐dose methotrexate/ifosfamide, etoposide, high‐dose cytarabine; DA‐EPOCH, dose‐adjusted etoposide, prednisone, vincristine, cyclophosphamide and doxorubicin; ECOG, Eastern Cooperative Oncology Group; Hyper‐CVAD, hyperfractionated cyclophosphamide, vincristine, doxorubicin and dexamethasone, alternating with high‐dose cytarabine and methotrexate; NNRTI, non‐nucleoside reverse transcriptase inhibitors; NRTI, nucleoside reverse transcriptase inhibitors; R‐CHOP, rituximab, cyclophosphamide, doxorubicin, vincristine and prednisone.

**FIGURE 1:**
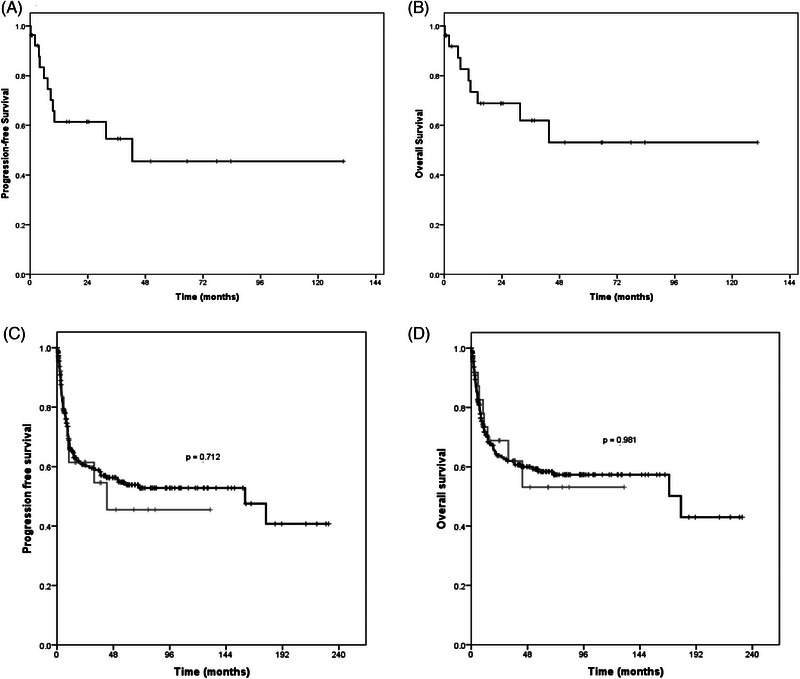
(A) Progression‐free survival; (B) overall survival; (C) progression‐free survival probability analysis comparing patients with immune reconstitution inflammatory syndrome (IRIS)‐associated lymphoma (grey line) and those with non‐IRIS‐associated lymphoma (black line); (D) overall survival probability analysis comparing patients with IRIS‐associated lymphoma (grey line) and those with non‐IRIS‐associated lymphoma (black line).

Four patients with IRIS‐associated lymphoma (14.8%) had primary refractory disease. Six patients (22.4%) died. Mean progression‐free survival was 67.6 months (95% confidence interval [CI]: 40.8–94.4) and mean overall survival was 77.6 months (95% CI: 51.1–104.1) (Figure 1A,B). In a comparative analysis with non‐IRIS‐associated lymphoma, progression‐free survival and overall survival were similar in the two groups (Figure 1C,D).

## 4 DISCUSSION

Immune recovery is a major goal of cART. However, as an exacerbated inflammatory response, IRIS leads to a paradoxical worsening in pre‐existing conditions, particularly infectious diseases. Although self‐limiting, it can be life threatening [1, 3].

IRIS‐associated conditions include fungal, viral and bacterial infections, cancer and autoimmune diseases. An established risk factor is severe immunosuppression preceding treatment (CD4‐lymphocyte counts < 100 cells/mm^3^) [1, 3].

In this cohort, prior to initiating cART, CD4 count was <200 cells/mm^3^ in 21 cases (77.7%), 200–350 cells/mm^3^ in two cases (7.4%) and >350 cells/mm^3^ in four cases (14.8%). The incidence of IRIS‐associated HL was reported to be high in patients with CD4 counts <350 cells/mm^3^ prior to initiating cART and even higher in patients with counts of 200–350 cells/mm^3^ [8].

At diagnosis of lymphoma, 16 patients (59.2%) had CD4 counts <200 cells/mm^3^. Kowalkowski et al. found that 45.2% patients with IRIS‐associated HL had CD4 counts <200 cells/mm^3^. A multicentre study that assessed cancer incidence after cART initiation in 11,485 patients reported a median CD4 count of 202 cells/mm^3^ (range 61–338 cells/mm^3^) at the moment of cancer diagnosis [9]. A more exacerbated immune response after starting cART seems to favour the occurrence of HL in this immunosuppressed population [5].

Gopal et al. evaluated 482 HIV‐infected patients and found an incidence of IRIS‐associated lymphoma of 12%, with a 49% overall survival rate [3]. In another study, the incidence of IRIS‐associated lymphoma was 8.6%, predominantly among males (89.7%) [10]. Similarly, the present incidence of IRIS‐associated lymphoma was 9.8%, with 85% of cases being in males. Likewise, Yanik et al. reported a prevalence of 79% in males and a median age of 38 years (range 32–45 years) [9].

The physiopathology of IRIS‐associated lymphoma is complex and poorly understood. Lanoy et al. first reported that initiating cART could precipitate the development of HL because of the sharp decrease in HIV viral load within 4 weeks of treatment [11]. They also reported the involvement of cytokines, such as CCL17 and CCL22, in the physiopathology of IRIS‐associated HL and NHL. Therefore, co‐infection with the Epstein–Barr virus (EBV) should be evaluated. Overexpression of the EBV oncoprotein, latent membrane protein‐1, in infected germinal centre cells during EBV infection is known to have a direct association with cell replication and oncogenesis. Therefore, IRIS‐associated lymphoma is a consequence of the complex association between viral oncogenes such as those of HIV and EBV, activation of signalling pathways, such as nuclear factor κ‐B, the interaction of tumour cells with the microenvironment and chronic B‐cell activation that varies according to histologic subtypes [9, 10, 11]. Likewise, co‐infection with hepatitis B or C viruses increases the risk of cancer in patients who initiate cART [12]. In the present study, however, there were no cases of IRIS‐associated lymphoma linked to the aforementioned co‐infections.

Regarding HIV viral load at diagnosis of lymphoma, in the present study 59.3% patients had <500 copies/mL, while 11.1% had 500–4999 copies/mL, 18.5% had 5000–49,000 copies/mL and 11.1% had >50,000 copies/mL. Bohlius et al. reported viral loads of 500–9999 copies/mL in 10% cases of HL after initiation of cART, 10,000–99,999 copies/mL in 27% cases, 100,000–499,000 copies/mL in 28% cases and ≥500,000 copies/mL in 12% cases [13].

In the present study, the time between HIV diagnosis and cART initiation was <12 months in 70.3% of cases. Conversely, Kowalkowski et al. reported that cART was initiated within 60 months of HIV diagnosis in 82% of cases [8]. There was no incidence peak in IRIS‐associated lymphoma over the entire period evaluated; therefore, it is impossible to speculate whether changes in the cART regimen, such as incorporating integrase inhibitors, would reflect on the reported incidence.

In our study, the time between cART initiation and lymphoma diagnosis was <3 months in 40.7% cases and 3–6 months in 59.3% cases. Kowalkowski et al. reported a higher incidence of IRIS‐associated lymphoma within 12 months of initiating cART, with a threefold greater risk in the first trimester [8]. Other studies corroborated these findings [14, 15].

In the present study, four patients (14.8%) were primary refractory to the established treatment and six patients (22.4%) died. Mean progression‐free survival was 67.8 months and mean overall survival was 77.6 months (Figure 1), with no significant difference when cases of IRIS‐associated lymphoma and non‐IRIS‐associated lymphoma were compared, corroborating the findings of Gopal et al. [3].

Limitations of the present study include its retrospective design. Furthermore, the patient records had been registered manually and were sometimes missing. No supplementary data on biopsies or records regarding EBV infection were available. The incidence density could not be determined due to the lack of data in the centres involved.

## 5 CONCLUSIONS

Despite the limitations, these findings are consistent with previous reports and relevant in view of the scarcity of studies on IRIS‐associated lymphoma.

## AUTHOR CONTRIBUTIONS

All authors were involved in the direct care of the patients and in interpreting medical tests. Juliano Cordova Vargas was responsible for the retrospective collection, analysis and interpretation of data. Otavio C. G. Baiocchi and Paulo Roberto Abrão Ferreira collaborated in writing the first draft of the manuscript. All the authors read and approved the final manuscript.

## CONFLICT OF INTEREST STATEMENT

The authors declare they have no conflicts of interest.

## FUNDING INFORMATION

The authors received no specific funding for this work.

## ETHICS STATEMENT

The internal review boards of the Federal University of São Paulo (approval #2.186.634), the Hospital Israelita Albert Einstein (#2.801.544), the São Paulo State Cancer Institute (#3.084.132), the Emílio Ribas Institute of Infectious Diseases (#2.753.893) and the Centro Paulista de Oncologia (#3.912.660) approved the study protocol.

## PATIENT CONSENT STATEMENT

The authors have confirmed patient consent statement is not needed for this submission.

## CLINICAL TRIAL REGISTRATION

The authors have confirmed clinical trial registration is not needed for this submission.

## Data Availability

The datasets analysed during the current study are available from the corresponding author upon reasonable request.
